# Exploring factors affecting owners’ trust of contractors in construction projects: a case of China

**DOI:** 10.1186/s40064-016-3393-9

**Published:** 2016-10-13

**Authors:** Shuangliang Tai, Chengshuang Sun, Shoujian Zhang

**Affiliations:** School of Civil Engineering, Harbin Institute of Technology, Harbin, 150001 China

**Keywords:** China, Trust attributes, Construction projects, PCA

## Abstract

**Background:**

It has been found that a low level of trust among members of a construction project team leads to poor performance in China. Many researchers have described the challenges, consequently advocating partnering as an attractive approach for more valuable cooperation. Because substantial investments have been poured into construction projects since the year 2000, trust research will improve the performance of construction projects and will be meaningful to the Chinese construction industry.

**Purpose:**

The purpose of this paper is to investigate the attributes affecting owners’ trust of contractors, to understand the potential properties of these factors, and to rank the factors in order of importance.

**Results:**

Twenty-four attributes are identified from a literature review. Supported by qualitative reviews, a questionnaire is conducted to obtain relevant data, and 168 valid responses are obtained for data analysis. Principal component analysis (PCA) is employed to find the factor structure of the identified trust attributes. By the method of PCA, the attributes are extracted into eight factors, including interaction history, information sharing and communication, contract and institution, relation-specific investment, reputation, integrity, competence, and opportunistic behaviour.

**Conclusions:**

The value and originality of this paper are embodied in using PCA to understand the various attribute groupings and to illuminate trust impact factors in the Chinese context. When they understand the critical factors affecting trust better, owners and contractors can devise more appropriate strategies to improve performance.

**Electronic supplementary material:**

The online version of this article (doi:10.1186/s40064-016-3393-9) contains supplementary material, which is available to authorized users.

## Background

Many studies have identified the importance of trust in construction projects (Wong et al. 2000, 2008; Pinto et al. 2009; Cheung et al. 2011). However, the construction industry has a poor record of trust among construction project team members due to uncertainties and fragmentations of the construction process (Karlsen et al. 2008). Since the middle of the 1990s, the construction industry has been strongly criticised by professionals for its poor performance and non-cooperative working atmosphere that is characterised by mistrust and conflict. Many studies have described the challenges of construction projects, thus advocating partnering as an attractive approach for more valuable cooperation (Ng et al. 2002; Bayliss et al. 2004; Cheung et al. 2012). One of the preconditions for the success of construction partnering is to build and develop trust among project team members. Trust and commitment is a critical factor for success of construction partnering (Cheung et al. 2003).

The construction industry represents one pillar in China’s national economy. However, construction projects in China suffer from cost overruns and delays frequently. This poor performance arises in part from a lack of trust between owners and contractors (Yun and Jiang 2010). In fact, distrust and suspicion between the two are very common during the construction stage of projects in China. Lack of trust leads to poor communications and an adversarial relationship. To some extent, owners and contractors play a zero-sum game in China (Yang and Shuai 2011). However, professionals are making every effort to improve the performance of China’s construction industry, and much attention has been given to trust among construction project team members both in practical and academic circles (Jiang et al. 2011).

Although much has been learned on this topic, little can be found about the factors affecting owners’ trust of contractors in China, the two most important players in the construction project team. Trust between these two parties is crucial to the success of the construction project team. In this regard, it is critical to explore the relative importance and groupings of the factors for trust in construction projects within the Chinese context. The goals of the paper are to investigate the influence factors affecting owners’ trust of contractors, to explore the potential properties of the factors, and to rank these factors in order of importance in China. An exploratory approach was adopted for this investigation. First, the list of attributes that influence trust was obtained by a literature review. Then, a questionnaire survey was conducted to collect quantitative data from owners using the attributes. The data collected was analyzed by PCA, which was employed to find the trust impact factor structure among the identified attributes and to get their groups. If they understand the critical factors affecting trust better, owners and contractors can formulate more appropriate strategies.

## An overview of trust and trust factors in construction

There are three parts in this section. Firstly, concepts of trust in the literature are summarised, and trust between owners and contractors in this paper is defined. Secondly, sources of trust are identified. Lastly, the attributes affecting owners’ trust of contractors are drawn from literature.

### Concept of trust

Trust is widely discussed in many disciplines from different perspectives. Many concepts of trust have been introduced in the literature. Sabel (1993) proposed that “trust is the mutual confidence that no party to an exchange will exploit another’s vulnerabilities”. Rousseau et al. (1998) defined trust as “a psychological state comprising the intention to accept vulnerability based upon positive expectations of the intentions or behaviour of another”. From the definition, trust is a state of mind that may lead to trusting intentions and behaviours. According to Nooteboom et al. (1997), trust is defined as “an expectation that people will not fail us, or the neglect or lack of awareness of the possibility of failure, even if there are perceived opportunities and incentives for it”.

We can extract three common elements of trust from the definitions. The first trust element is uncertainty about the future. The second involves vulnerability, that is, the risk of loss. The third involves control: trust is placed in another who is not under one’s control.

Trust concerns trusting intentions and trusting beliefs. A trusting intention implies a willingness to become vulnerable to another in a risky situation, while a trusting belief is the expectation that one will not be harmed by another in the risky situation (McKnight et al. 2002). Both factors are affected by one’s own inclination to trust. Therefore, a distinction can be made between trustworthiness on the part of the trustee, and trust on the side of the trustor (McEvily et al. 2003).

As Gambetta (1998) observed, “trust is related to the limits of our capacity ever to achieve full knowledge of others, their responses and their motives to endogenous as well as exogenous changes”. Therefore, many researchers have connected trust with risk (Nooteboom et al. 1997; Das and Teng 1998).

In the construction industry, owners lead the relationship with contractors. Therefore, trust in this paper is defined as an expectation of owners that the contractors will not conduct opportunistic behaviours, even when facing opportunities for get short-term gains by doing so (Laan et al. 2011).

### Sources of trust

Because trust is defined as an expectation that a contractor will not fail an owner, we can question whether the contractor who is trusted has the capability to meet the expectation. This aspect of trust is called competence trust in the literature. It shows the level of trust one has in the managerial, organisational, and technical competences of the trustee (Nooteboom et al. 1997).

Another question is whether the contractor intends to use their capability to meet to the owner’s expectations (Laan et al. 2011). This aspect of trust is the intentional aspect of trust. It refers to the intentions of the contractor towards the relationship. Intentional trust can be divided into trust in benevolence and trust in dedication, where benevolence represents the willingness to positively treat the owner under unforeseen circumstances and dedication represents the willingness to employ one’s capabilities in the relationship (McAllister 1995).

Researchers in economics argue that trust comes from rational calculation, emphasising the extrinsic value of trust. However, researchers in psychology argue that trust comes from a social orientation towards other people, emphasising the intrinsic value of trust (e.g., Rousseau et al. 1998; Nooteboom et al. 1997). Nooteboom et al. (1997) assumed that trust has both rational reasons and psychological causes. The rational reasons for trust are based on the trustee’s trustworthiness, while its psychological causes are effects, routine, the neglect of relational risk, etc. (Laan et al. 2011).

Therefore, competence and the intentions of the contractor are primary sources of trust between the contractor and the owner.

### Attributes affecting trust in construction

The attributes affecting trust in a construction project team have been studied by many researchers worldwide. To identify as many of the publications about trust research in construction projects as possible and provide a comprehensive review on the subject, Google Scholar, EI Compendex, and Web of Science were searched using the keywords “trust”, “construction project”, or “construction industry”. In total, 45 publications on trust in construction were identified for further analysis. The publications consist of journals papers, conference papers, theses, and some chapters in books. Sixteen of the publications were in Chinese.

These publications were reviewed to theoretically derive trust attributes in construction projects. The literature review indicated that many attributes had been identified as important for trust in construction projects. Based on the literature review, twenty-four trust attributes were identified, see Table 1.

**Table 1:** Trust attributes from the literature (the following attributes have a high impact on trust based on literature review)

Attributes	Frequency	Examples
C1 competence	15	Fong and Lung (2007), Kadefors (2004) and Cheung et al. (2003)
C2 honesty	12	Hartman (2015), Yeung et al. (2007) and Wang (2008)
C3 problem solving mechanism	6	Khalfan et al. (2007), Fong and Lung (2007) and Yeung et al. (2007)
C4 similarity	5	Nath and Mukherjee (2003) and Kadefors (2004)
C5 information sharing	9	Fong and Lung (2007), Hartman (2015) and Yeung et al. (2007)
C6 promise keeping	8	Wood et al. (2002) and Wong et al. (2010)
C7 reputation	8	Fong and Lung (2007) and Yeung et al. (2007)
C8 mutual respect	4	Yeung et al. (2007) and Cheung et al. (2003)
C9 long-term cooperation	3	Yeung et al. (2007) and Smyth and Edkins (2007)
C10 fairness	4	Wong and Cheung (2005) and Yeung et al. (2007)
C11 effective communication	11	Wood et al. (2002) and Karlsen (2008)
C12 frequent communication	11	Fong and Lung (2007), Yeung et al. (2007) and Kadefors (2004)
C13 consistency between efforts and rewards	8	Wood et al. (2002) and Cheung et al. (2003)
C14 behaviour reliability	6	Cheung et al. (2003) and Karlsen (2008)
C15 confidence in the other	5	Smyth and Edkins (2007) and Atkinson et al. (2006)
C16 completeness of contract	10	Wong and Cheung (2005), Fong and Lung (2007) and Yeung et al. (2007)
C17 opportunistic behaviour	4	Jannadia et al. (2000) and Seymour and Rooke (1995)
C18 supervision of the third party	2	Cheung et al. (2003)
C19 goal achievement	3	Karlsen (2008) and Cheung et al. (2003)
C20 interaction experience	3	Khalfan et al. (2007), Hartman (2015)
C21 sense of social responsibility	2	Karlsen (2008)
C22 good intentions	4	Karlsen (2008), Wong et al. (2010)
C23 common goal	5	Khalfan et al. (2007), Karlsen (2008)
C24 mutual interdependence	4	Khalfan et al. (2007), Wood et al. (2002)

Not all of the literature is listed in the table due to limited space

## Methods

In this study, a questionnaire survey was employed to collect quantitative data from owners. The methods of questionnaire is frequently employed by researchers to collect information about opinions and attitudes. Therefore, the method is suitable for trust research in this paper.

The target participants that were selected are owners since they lead the relationship with contractors. The questionnaire consists of two parts. Part I includes the profile of the participants, and Part II includes scales to measure the influence of 24 attributes on trust (as shown in Additional file 1). Some of the preliminary measurements are adapted from literature in English and translated into Chinese. The other measurements are from field interviews or literature in Chinese. Next, the preliminary measurements were modified by experienced consulting professionals and academics so that they were accurate, concise and clear.

The questionnaire was sent to the targeted participants by e-mail through the China Construction Industry Association (CCIA). For each of the 24 attributes, the participants were asked to state whether they agreed that the attribute has a high impact on trust in construction projects using a 5-point Likert scale (1 = Strongly Disagree and 5 = Strongly Agree).

PCA was employed to find groupings of the 24 identified variables and to investigate the potential characteristics. The goal of PCA is to find the crucial information from the data and to show the information as a set of new orthogonal variables, the principal components (Abdi and Williams 2010). Normally, there are fewer principal components than original variables. The principal components obtained are ranked according to the variance explained. The independence of the principal components is assured if the data set is jointly normally distributed. Cronbach’s reliability test is used to determine the reliability of the attributes (Chiu and Lau 2015). Typically, a scale with a threshold value of .70 is regarded as having acceptable internal consistency (Wai et al. 2013).

To understand properties of identified factors better, semi-structured interviews with questions about trust were conducted with experienced practitioners.

## Results

### Introduction to the survey

We conducted the questionnaire survey from December 2012 to March 2013. All of the participants are project executives representing owners. One hundred and eighty-two replies out of 312 questionnaires were collected, and 168 replies were valid. The target construction projects were located in Changsha, Harbin, Guangzhou, Chongqing, and Beijing.

The profiles of participants are summarised in Table 2. The first two rows are information about the participants’ firms, and the last two rows are information about the participants’ current projects.

**Table 2:** Profiles of the participants

Profile	Category	Percentage
Ownership of the firm	Public agencies	35.1
	State owned firms	41.4
	Private firms	23.5
Number of employees	<500	30.6
	500–2000	47.8
	More than 2000	21.6
Type of project	Industrial buildings	10.4
	Commercial buildings	7.8
	Residential buildings	43.8
	Public buildings	4.1
	Infrastructure projects	30.1
	Others	3.8
Duration of project	<1 year	8.1
	1–3 years	69.6
	More than 3 years	22.3

### Data tests

Before the analysis, we have to make sure the reliability of the trust attributes and the adequacy of the sample size. SPSS 16.0 was employed for analysis in this study.

The results for the reliability of attributes are presented in Table 3. The alpha value is .892 so that the reliability of the 24 attributes is enough for factor analysis.

**Table 3:** Reliability Statistics for the 24 Attributes

Cronbach’s alpha	Cronbach’s alpha based on standardised items	No of items
.892	.896	24

Then, Kaiser–Meyer–Olkin (KMO) test and Bartlett’s Test of Sphericity were conducted. The KMO is expressed as an index ranging from 0 to 1. The literature suggests that the value of KMO should be greater than .70 for factor analysis (Stern 2009). Bartlett’s Test of Sphericity checks the probability that the matrix is an identity matrix (Hair et al. 2006). The significance level of less than .05 is regarded as suitable. The value of KMO test is .741 and Bartlett’s Test of Sphericity is also significant (see Table 4). Therefore, the data is suitable for PCA.

**Table 4:** KMO and Bartlett tests

Kaiser–Meyer–Olkin sampling adequacy	.741
Bartlett’s test of sphericity	
Approx. Chi square	885.345
*df*	184
Sig.	.000

### Extraction of factors

After all of the necessary requirements are satisfied, we conducted the factor analysis by PCA with varimax rotation. The rotation method is employed because it maximises the variance of the factor loadings by making the high loadings higher and the low loadings lower on each factor (Chiu and Lau 2015). The eigenvalue is used to measure the contributions of a variable to a principal components. Generally, extracted components with eigenvalues equal to or greater than 1.0 are regarded as being significant factors (Stern 2009).

A clear component structure exists if a variable has a significant factor loading (loading > 0.50) on one component only (Spector 1992). The loading is employed to represent the impact of each variable on the individual component (Chiu and Lau 2015).

The rotated component matrix is shown in Table 5. The accumulated variance of the components is shown in Table 6. We extracted eight components when we applied the rule of an eigenvalue greater than 1.0. The cumulative total variance accounted for 69.031 % of the variation, which satisfies the criterion that factors should explain at least 50 % of the variation. Table 5 shows that each attribute has significant loading on only one component and more than one variable load on each component. Therefore, all eight components can be kept for further analysis.

**Table 5:** Rotated component matrix

Attributes	Components							
	1	2	3	4	5	6	7	8
C4	*.785*	.045	.106	.176	.186	−.065	.186	−.082
C9	*.687*	.074	.106	.193	.035	−.053	.174	−.056
C20	*.634*	.145	.201	.083	.021	.042	.176	.054
C23	*.625*	.065	.173	.186	.079	−.066	.174	−.073
C3	.043	*.768*	.108	.073	.115	−.022	.187	.067
C10	.156	*.701*	.175	.056	.085	.052	.178	.062
C16	.175	*.624*	.193	.098	.137	−.064	.165	.042
C18	.105	*.619*	.078	.073	.124	−.032	.154	.043
C5	.175	.024	*.723*	.067	.184	.196	.043	−.023
C11	.067	.093	*.703*	.162	.046	.187	.101	−.012
C12	.087	.165	*.693*	.201	.189	.054	.123	.012
C8	.137	.174	.108	*.724*	.164	.043	.186	−.043
C22	.142	−.176	.065	*.701*	.045	.097	.054	−.065
C24	.107	.153	.063	*.684*	.143	.042	.195	−.062
C1	.164	.197	.064	.095	*.653*	−.083	.198	−.045
C15	−.021	.065	.045	−.043	*.645*	.058	.165	−.073
C19	.043	.172	.190	.044	*.603*	−.106	.045	−.051
C7	.097	−.043	.021	−.032	.021	*.735*	.053	.103
C13	.056	.074	.043	.164	.075	*.611*	.198	−.021
C21	.032	.179	.154	.011	.102	*.606*	.221	−.032
C2	.143	−.105	.092	.235	.194	.083	*.802*	−.023
C6	.052	−.064	−.076	.014	.107	.074	*.734*	.078
C14	.136	.056	.076	.124	.201	.153	*.672*	.013
C17	−.032	.012	−.032	−.065	−.054	.154	−.021	*.813*

**Table 6:** Accumulated variance explained by the components

Accumulated variance		
Component	% of variance	Cumulative %
1	16.924	16.924
2	10.429	27.353
3	8.713	36.066
4	7.536	43.602
5	7.177	50.779
6	6.901	57.681
7	6.329	64.010
8	5.021	69.031

Followings are to explain the eight principal components extracted. Since the paper employs an exploratory approach involving many attributes, the explanation of the components is challenging. Another challenge is that the combinations of variables that load high on a component have patterns that are difficult to interpret. Therefore, the explanation of factors needs a certain amount of imagination and ingenuity (Wai et al. 2013). In the following section, based on the content and relationships among the variables, the labelling and explanation of each component is given.

## Discussions

After a critical inspection of variables in the components, a proper name is given to each extracted factor. The eight factors are named as follows: the interaction history factor, the contracts and institutions factor, the information sharing and communications factor, the relation specific building factor, the competence factor, the reputation factor, the integrity factor, and the opportunistic behaviours factor.

Based on the findings from the survey and the opinions from interviews, the meanings of the eight factors are explained as follows.

### Interaction history

The first factor includes four variables, namely “long-term cooperation”, “interaction experience”, “similarity”, and “common goals” (see Table 6). These four variables explain 16.924 % of the variance. Based on the potential characteristics of these variables, we name the factor as “interaction history”.

Because of the dynamic, challenging, and complex nature of construction projects, interaction history is very important. For instance, relational bonding is identified as one of the trust factors because it explains trust between organisations derived from repeated interactions over time (Kadefors 2004).

The Chinese construction industry has blossomed since the year of 2000, which has created many contracting opportunities in construction market. Therefore, behaving trustworthy in past projects provides more opportunities for future projects.

The results from semi-structured interview also confirm the findings, and most of the participants noted that interaction history is very significant, especially long-term cooperation. Long-term interaction experience can significantly reduce uncertainties in terms of the other party’s behaviours and thus improve trust.

### Contracts and institutions

From Table 6, factor 2 explains 10.429 % of the total variance. The factor includes “fairness”, “mechanism for solving problems”, “third party supervision”, and “completeness of the contract”. We note that these variables concern the problem solving system, the rights and obligation allocation system, and third party supervision. It is straightforward to label this factor as “contracts and institutions factor”.

A contract with rigorous and comprehensive terms, a fair allocation of risks, an effective dispute resolution mechanism, and reliable supervision by independent agencies, is an institutional guarantee that protects the owners’ interests. Therefore, the contracts and institutions factor plays an important role in trust. Cheung et al. (2003) found that a clearly defined contract brings comfort and confidence to the owner and the contractor, which can promote trust building process. In addition, Wong and Cheung (2005) noted that problem-solving ability is a critical attribute for the development of trust. If a conflict is resolved friendly, trust will be built.

This factor is confirmed by the interviews. Some interviewees feel safer and more likely to trust the contractors if their interests are protected by a good contract and institutional arrangements.

### Information sharing and communication

Factor 3 consists of three variables, information sharing, frequent communication and effective communication, which account for 8.713 % of the total variance. This factor is labelled “information sharing and communication”. By exchanging of information and reducing misunderstandings, communication enables the parties to recognise mutual benefits. Information sharing can signal the willingness of a partner to be transparent, which is a key attribute of trustworthiness in a relationship (Wong and Cheung 2005).

The importance of frequent and effective communication and information sharing to increasing trust levels was confirmed by Wong and Cheung (2005). If clear and accurate information is disseminated in a timely manner, uncertainty will be reduced, thereby reducing misunderstand and enabling partners to work better together, finally improving the trust.

### Relation-specific investments

Factor 4 includes “good intentions”, “mutual respect”, and “mutual interdependence”, which account for 7.536 % of the total variance (see Table 6). Good intentions mean that one party cares about the interests of the other party. Mutual respect means that a party treats the other equally and respectfully. Mutual interdependence means that both sides need to maintain their relationship.

Heide and John (1988) argued that mutual interdependence between enterprises comes from a “relation-specific investment” in maintaining cooperation, including investment in fixed assets, training and databases. Gundlach et al. (1995) argued that relation-specific investment is actually a type of attitudinal commitment reflecting the willingness to maintain cooperative relations and mutual recognition. A typical feature of a relation-specific investment is high sunk costs. If the relationship is broken, it is difficult to recover its value.

To some extent, mutual respect, good intentions and mutual interdependence are the result of a relation specific investment. Therefore, this factor is called relation specific investments.

### Competence

Factor 5 consists of three variables: competence, confidence in the other, and goal achievement, which account for 7.177 % of the total variance (see Table 6). The competence of contractors means that they have the technological and managerial skills to complete the project strictly according to the contract. The literature shows that the competence of a trustee is a precondition of trust. When the owner perceives the contractor’s competence, it engenders confidence in the contractor. Higher competence means a greater likelihood of achieving the project goals. These three attributes are closely related to competence, so the factor is called competence.

### Reputation

Factor 6 consists of “consistency between efforts and rewards”, “reputation”, and “sense of social responsibility”, which account for 6.901 % of the total variance. Reputation is an opinion about a contractor that is typically the result of a social evaluation based on a set of criteria. Reputation is difficult to build but is easy to destroy. A reputable contractor always tends to behave ethically and in a trustworthy manner. If a contractor has high social responsibility, he cares about the interests and welfare of the public. Therefore, this factor is called reputation.

### Integrity

Factor 7 consists of three variables: “honesty”, “promise keeping”, and “behaviour reliability”, which account 6.329 % of the total variance. The factor is easy to interpret. If contractors always match their word with their deeds, they will be considered to be trustworthy.

### Opportunistic behaviours

Factor 8 includes only one variable: “opportunistic behaviour”. Opportunistic behaviour means that a contractor takes advantage of the owner’s weaknesses to improperly benefit. Obviously, opportunistic behaviour harms trust.

These factors can be classified into three different categories. Reputation factor, competence factor, promise-keeping factor, and opportunistic behaviours factor are attributes of the contractor. These attributes represent the premise and preconditions for establishing trust. Information sharing and communication factor, interaction history factor and relation-specific investment factor involve both parties and belong to the relational dimension. They are methods and channels for maintaining and improving trust. The contract and institution factor is an institutional aspect that guarantees trust establishment and maintenance.

## Conclusions

Trust between the owner and the contractor is currently an important concern in China. The poor performance of many construction projects has brought attention from construction professionals to know the key factors affecting trust. Taking a lead from previous studies, twenty-four attributes that affect trust are identified in this study, and the method of PCA is employed to obtain factor structure of the trust attributes. The 24 trust attributes are extracted into eight factors, including information sharing and communication, interaction history, contracts and institutions, competence, relation-specific investment, reputation, integrity, and opportunistic behaviours. These factors form basis for improving owners’ trust of contractors in China. The value and originality of this paper is to use PCA to group these various trust attributes and expound these eight trust factors for construction in China.

This paper has two contributions. Firstly, it is a context-driven research that identifies trust factors. Second, by grouping these factor components, the findings provide implications that will assist owners and contractors to improve the trust between them. The factor component groupings represent the critical trust attributes, implying that these eight factors should be considered carefully. The enhanced understanding presented in the paper enables the development of methods and strategies for building trust.

However, this research has some limitations. In terms of its methodological approach, this paper is a exploratory and confirmatory research. In addition, a larger sample size would have provided a more robust factor analysis. Future research to explore exactly how these trust impact factors can be enacted is important for improving trust between owners and contractors.

## Additional file

10.1186/s40064-016-3393-9 Questionnaire on Trust Factors between Owners and Contractors in China.
