# Supplementary material for: Exploring factors affecting owners’ trust of contractors in construction projects: a case of China
Source: Springerplus. 2016 Oct 13;5(1):1783. doi: 10.1186/s40064-016-3393-9 (PMC5063835; doi:10.1186/s40064-016-3393-9)
Supplement: Supplementary file 1 — 10.1186/s40064-016-3393-9 Questionnaire on Trust Factors between Owners and Contractors in China. [file 40064_2016_3393_MOESM1_ESM.docx]

**Questionnaire on Factors Affecting Owners’ trust on Contractors in China**

The questionnaire consists of two parts. Part I includes the profile of the participants. Part II includes measurements to measure the influence of the 24 attributes on trust.

Part I Profile of respondents

1 What is the ownership of your firm?

1. Public agency
2. State owned firm
3. Private firm

2 What is the number of employees in your firm?

1. Less than 500
2. 500-2000
3. More than 2000

3 What type of project is this?

1. Industrial building
2. Commercial building
3. Residential building
4. Public building
5. Infrastructure project
6. Other

4 What is the duration of the project?

1. Less than one year
2. 1-3 years
3. More than 3 years

Part II Trust factors

Please indicate whether you agree that these attributes have a high impact on trust in construction projects using the 5-point Likert scale, ranging from strongly disagree (1) to strongly agree (5).

| Attributes | Measurements /Explanations | Scale |
| --- | --- | --- |
| C1 | The contractor has skilled professional technology and a higher management level | 1 2 3 4 5 |
| C2 | The contractor is honest regarding execution or changes in construction projects | 1 2 3 4 5 |
| C3 | Problems can be solved through amicable negotiations | 1 2 3 4 5 |
| C4 | The contractor shares our values and behaviour patterns | 1 2 3 4 5 |
| C5 | The two parties have timely and effective information sharing | 1 2 3 4 5 |
| C6 | The contractor tries his/her best to fulfil his promises | 1 2 3 4 5 |
| C7 | The contractor has good reputation in the construction industry | 1 2 3 4 5 |
| C8 | The two parties are equal and no party is belittled | 1 2 3 4 5 |
| C9 | We have a long-term partnership with the contractor | 1 2 3 4 5 |
| C10 | Interests and risks are shared fairly and reasonably | 1 2 3 4 5 |
| C11 | The two parties communicate effectively and each knows the needs of the other | 1 2 3 4 5 |
| C12 | The two parties have frequent communications | 1 2 3 4 5 |
| C13 | There is consistency between efforts and rewards | 1 2 3 4 5 |
| C14 | The contractor behaves reliably and can meet our expectations | 1 2 3 4 5 |
| C15 | We have confidence in the contractor's competence | 1 2 3 4 5 |
| C16 | The rights and obligations of each party are clearly and completely expressed in a contract | 1 2 3 4 5 |
| C17 | The contractor will not take advantage of our weaknesses (such as an incomplete contract or information asymmetry) | 1 2 3 4 5 |
| C18 | A consulting firm evaluates the contractor | 1 2 3 4 5 |
| C19 | Project goals can be satisfactorily achieved | 1 2 3 4 5 |
| C20 | We have experienced successful cooperation with the contractor | 1 2 3 4 5 |
| C21 | The contractor has strong sense of social responsibility and receives public praise | 1 2 3 4 5 |
| C22 | The contractor is friendly and cares about our interests | 1 2 3 4 5 |
| C23 | The two parties have common goals at every stage of construction | 1 2 3 4 5 |
| C24 | The contractor inputs many resources to maintain a good relationship with us | 1 2 3 4 5 |
